# Hypertension of liver-yang hyperactivity syndrome induced by a high salt diet by altering components of the gut microbiota associated with the glutamate/GABA-glutamine cycle

**DOI:** 10.3389/fnut.2022.964273

**Published:** 2022-08-09

**Authors:** Tao Zheng, Yi Wu, Mai-jiao Peng, Nen-qun Xiao, Zhou-jin Tan, Tao Yang

**Affiliations:** ^1^College of Food Science and Engineering, Central South University of Forestry and Technology, Changsha, China; ^2^School of Pharmacy, Hunan University of Chinese Medicine, Changsha, China; ^3^Medical School, Hunan University of Chinese Medicine, Changsha, China

**Keywords:** high salt diet, hypertension, liver-yang hyperactivity syndrome, gut microbiota, metabolite, glutamate/GABA-glutamine cycle

## Abstract

The gut microbiota and metabolites are closely related to hypertension; however, the changes in the composition of the gut microbiome and metabolites linking a high salt diet to elevated blood pressure are not established. In this study, traditional Chinese medicine (TCM) syndrome of hypertension caused by high salt had been diagnosed and the pathogenesis of hypertension was explored from the perspective of intestinal microecology. Rats in a high salt diet-induced hypertension group (CG) and normal group (CZ) were compared by 16S rRNA gene full-length sequencing and liquid chromatography and mass spectrometry to identify differences in the bacterial community structure, metabolites, and metabolic pathways. Hypertension induced by a high salt diet belongs to liver-Yang hyperactivity syndrome. Alpha and beta diversity as well as the composition of microbiota from the phylum to species levels differed substantially between the CG and CZ groups. In an analysis of differential metabolites in the intestines, a high salt diet mainly affected the metabolism of amino acids and their derivatives; in particular, γ-aminobutyric acid (GABA) was down-regulated and glutamic acid and its derivatives were up-regulated under a high salt diet. Based on a KEGG analysis, high salt intake mainly altered pathways related to GABA and the glutamate/glutamine metabolism, such as the GABAergic synapse pathway and glutamatergic synapse pathway. The correlation analysis of differential gut microbes and differential metabolites suggested that a high salt diet promoted hypertension *via* the inhibition of Clostridiaceae_1 growth and alterations in the GABA metabolic pathway, leading to increased blood pressure. These findings suggest that a high salt diet induces hypertension of liver-Yang hyperactivity syndrome by mediating the microbiota associated with the glutamate/GABA-glutamine metabolic cycle *via* the gut–brain axis.

## Graphical Abstract

The process of animal experimental verification. 
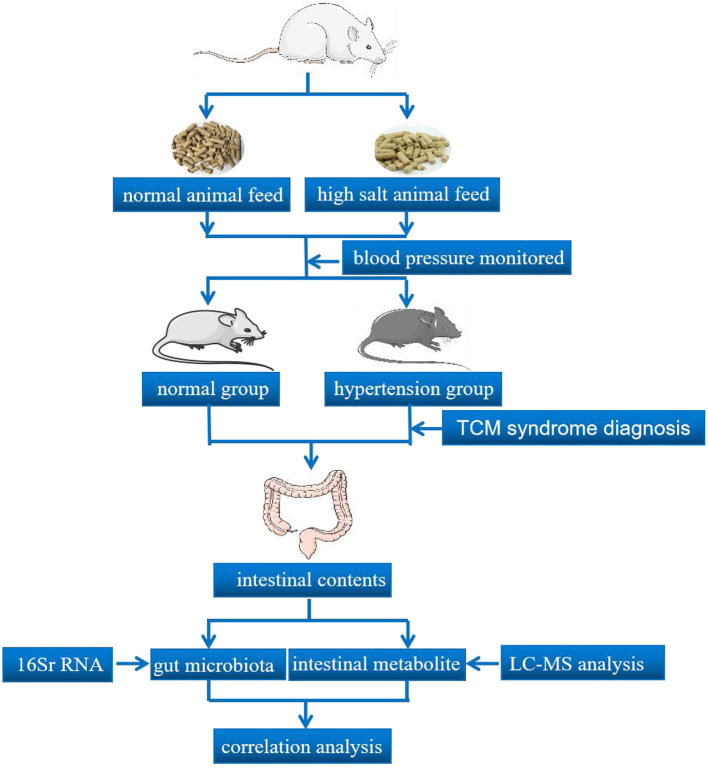

## Introduction

Hypertension is a key contributor to cardiovascular disease and a major risk factor for morbidity and mortality worldwide (1). Hypertension is a complex disease characterized by persistently elevated blood pressure in the arteries, and damage to the kidneys, brain, and vascular system could increase the risk of hypertension (2). However, the pathogenesis of hypertension is not fully understood. Thus, elucidating the pathogenesis and identifying new strategies for the treatment of hypertension will have a significant impact on human health and wellbeing. The diagnosis and treatment of hypertension with TCM had the advantages of individualized and precise treatment and small side effects (3). Experts of Society of cardiovascular diseases, China association of Chinese medicine consensus on diagnosis and treatment of hypertension with traditional Chinese medicine and they standardized the identification of hypertension syndrome type and the use of prescription (4).

Salt is an essential micronutrient that enhances taste and is widely used in the food industry as a preservative. Owing to dietary habits and trends in the food industry, salt consumption is far more than the recommended 6 g/day limit and the threat of health issues due to a high salt diet is becoming increasingly apparent (5). In clinical research on hypertension, a high salt diet is an important cause, and daily sodium intake is positively associated with the aggravation of hypertension (6, 7). Epidemiological studies have also confirmed that salt intake is closely associated with increased blood pressure (8–10).

The gut microbiota has been recognized as an important and independent metabolic organ. The gut microbiota and its metabolites are considered key regulators of brain–gut function (11) and have attracted widespread attention owing to associations with chronic diseases (12). Diet affects the composition of the gut microbiota and dysbiosis of gut microbiota has been found in both patients with hypertension and preclinical models (13–16). Salt intake affects the gut microbiota, microbial metabolite composition, and blood pressure (17). Traditional Chinese medicine (TCM) syndrome of hypertension caused by high salt had been diagnosis and we characterized changes in the gut microbiota and metabolites induced by a high salt diet and evaluated correlations between microbial alterations and differential metabolites. These results provide an insight into the pathogenesis of high salt diet-induced hypertension. Correlation analysis of gut microbiota, metabolites and related pathways can provide a theoretical basis for the treatment of hypertension in TCM from the perspective of intestinal microecology, and can guide people to use dietary salt rationally.

## Materials and methods

### Animals

Male Wistar rats (3 to 4 weeks old, 55–75 g) were purchased from Beijing Vital River Laboratory Animal Technology Co., Ltd. (Beijing, China), with license number SCXK (Jing) 2016-0006. Wistar rats were raised in a shielded environment at the Animal Experiment Center of the Hunan University of Chinese Medicine with license number SYXK (Xiang) 2015-0003 under a 12/12 dark–light cycle (21 ± 2°C with a relatively constant humidity of 45 ± 10%). Mice were provided ad libitum access to food and water.

### Hypertension modeling induced by a high salt diet

Twelve Wistar rats were randomly divided into a normal group (CZ group) and hypertension model group (CG group) (six rats per group). Rats in the CG group were given 8% high salt animal feed and those in the CZ group were given normal animal feed. High salt feed and normal feed were obtained from the same feed company (Beijing Vital River Laboratory Animal Technology Co., Ltd.) and had the same nutrient contents, except for the salt content. In the analysis of the development of hypertension, blood pressure was measured by rat caudal artery manometry once a week.

### Intestinal content collection

The intestinal tract tissue from the pylorus of the stomach to the ileocecus was cut longitudinally with sterile scissors to peel away the contents. Samples of the intestinal contents in the CZ group and CG group were collected in sterilized tubes and stored at −80°C. For the CZ and CG groups, four samples from each group were selected for gut microbiota detection by 16S rRNA gene sequencing and five samples were selected for intestinal metabolite detection by UPLC-MS.

### 16S rRNA PacBio SMRT gene full-length sequencing

PacBio SMRT sequencing technology was used to accurately obtain full-length 16S rRNA gene sequences (18, 19). Total microbial genomic DNAs of intestinal contents samples were extracted following the manufacturer's instructions and stored at −20°C. Total microbial genomic DNA samples were extracted using the OMEGA DNA Isolation Kit (D5625-01; Omega, Knoxville, TN, USA) following the manufacturer's instructions. The DNA concentration was determined using the NanoDrop ND-1000 spectrophotometer (Thermo Fisher Scientific, Waltham, MA, USA). PCR amplification of the nearly full-length bacterial 16S rRNA gene was performed using the forward primer 27F (5′-AGAGTTTGATCMTGGCTCAG-3′) and the reverse primer 1492R (5′-ACCTTGTTACGACTT-3′). The extracted DNA was amplified by two-step PCR, with sample-specific 16 bp barcodes incorporated into the forward and reverse primers for multiplex sequencing in the second PCR step. Next, the TruSeq Nano DNA LT Library Prep Kit was used to prepare the sequencing library. The library was tested using the Agilent High Sensitivity DNA Kit on the Agilent Bioanalyzer (Santa Clara, CA, USA). Finally, the amplified DNA fragment was sequenced using the MiSeq sequencer to obtain 2 × 300 bp paired-end reads with the MiSeq Reagent Kit V3 (600 cycles). SMRT sequencing technology and the PacBio Sequel platform were used for analyses at Shanghai Personal Biotechnology Co., Ltd. (Shanghai, China).

### Bioinformatics and statistical analyses

All fastq files were subjected to quality control using the QIIME 1.9.1 workflow. The remaining high-quality sequences were clustered into operational taxonomic units (OTUs) at a 97% sequence identity threshold using UCLUST (20). The Biological Observation Matrix (BIOM) file was used for the downstream analysis using the QIIME 1.9.1 pipeline and R language (v3.2.0). Alpha diversity was measured based on the number of observed OTUs and Chao1, Simpson, ACE, and Shannon indices rarified at the same sequencing depth. Beta diversity was analyzed to investigate structural variation in microbial communities across samples based on UniFrac distance metrics and a principal coordinate analysis (PCoA). The Wilcoxon rank sum test was used to analyze differences in the relative abundance of microbial taxa. Linear discriminant analysis effect size (LEfSe) was performed to identify differences in the microbial structure between groups, with default parameters.

### Untargeted omics detection of intestinal metabolites by UPLC-MS and statistical analyses

#### Metabolite extraction

After 100 mg (±1%) of each sample was added to a 2 mL EP tube, 0.6 mL of 2-chlorophenylalanine (4 ppm) methanol (−20°C) was added and vortexed for 30 s (samples of less than 50 mg were extracted by half of the experimental system). Then, 100 mg of glass beads was added, placed in a tissue grinder, and ground for 90 s at 55 Hz. Samples were subjected to ultrasound treatment at room temperature (26°C) for 10 min and centrifugation at 12,000 rpm and 4°C for 10 min. Then, 200 μL of the supernatant was filtered through a 0.22 μm membrane, and the filtrate was added to a detection bottle. A volume of 20 μL was obtained from each sample for quality control (QC). (These QC samples were used to monitor deviations in the analytical results from pooled samples and errors caused by the analytical instrument itself.) Samples were then used for LC-MS detection following previously described methods (21–24).

#### Detection

Chromatographic conditions were as follows. Chromatographic separation was accomplished using the Thermo Ultimate 3000 system equipped with an ACQUITY UPLC® HSS T3 (150 × 2.1 mm, 1.8 μm; Waters, Milford, MA, USA) column maintained at 40°C. The temperature of the autosampler was 8°C. Gradient elution of analytes was carried out with 0.1% formic acid in water (C) and 0.1% formic acid in acetonitrile (D) or 5 mM ammonium formate in water (A) and acetonitrile (B) at a flow rate of 0.25 mL/min. Injection of 2 μL of each sample was done after equilibration. An increasing linear gradient of solvent B (v/v) was used as follows: 0–1 min, 2% B/D; 1–9 min, 2–50% B/D; 9–12 min, 50–98% B/D; 12–13.5 min, 98% B/D; 13.5–14 min, 98–2% B/D; 14–20 min, 2% D-positive model (14–17 min, 2% B-negative model).

Mass spectrometry conditions were as follows. The ESI-MS experiments were executed on the Thermo Q Exactive Focus mass spectrometer with a spray voltage of 3.5 kV and −2.5 kV in positive and negative modes, respectively. Sheath gas and auxiliary gas were set at 30 and 10 arbitrary units, respectively. The capillary temperature was 325°C. The Orbitrap analyzer scanned over a mass range of m/z 81–1000 for full scans at a mass resolution of 70,000. Data-dependent acquisition (DDA) MS/MS experiments were performed with HCD scans. The normalized collision energy was 30 eV. Dynamic exclusion was implemented to remove unnecessary information in MS/MS spectra (25). Variable importance in projection VIP (value importance in projection) ≥ 1, *P* ≤ 0.05, and one-way ANOVA *P* ≤ 0.05 were used as thresholds to identify metabolites with significant differences (biomarker). We performed an integrated metabolic pathway analysis using KEGG to evaluate candidate biomarkers.

### Correlations between the gut microbiota and metabolites

The correlation between the gut microbiota and metabolite profile was analyzed. Spearman's correlation coefficients were used to evaluate the correlations between the gut microbiota and the host metabolome.

### Statistical analysis

Results are presented as means ± SEM. Bar plots were generated and statistical analyses were performed using GraphPad Prism 7 by using one-way ANOVA and *t*-tests. *P* < 0.05 was considered significant. *P*-values are represented as follows: ^*^P < 0.05, ^**^P < 0.01, ^***^P < 0.001, and N.S., not significant (*P* > 0.05).

## Results

### Hypertension induced by a high salt diet and traditional chinese medicine (TCM) syndrome

As shown in Table 1, there were no significant differences in systolic blood pressure (SBP) or diastolic blood pressure (DBP) between the CZ and CG groups before the administration of a high-salt diet. From week one, SBP values in the CG group were significantly higher than those in the CZ group (*P* < 0.05). For DBP, from week three, DBP values in the CG group were significantly higher than those in the CZ group (*P* 0.05). At weeks 5 and 6, DBP and SBP values in the CG group were over 20% higher than those in the CZ group, and every rat in the CG group developed hypertension. These results suggested that Wistar rats in the experimental group developed hypertension induced by a high salt diet. According to the degree of hyperactivity and irritability, rotation tolerance and behavior test and other indicators, it could be determined that the hypertension induced by a high salt diet belonged to the liver -Yang hyperactivity syndrome.

**Table 1:** Blood pressure of Wistar rats with a high salt diet (mmHg).

**Time**	**CZ**		**CG**	
	**SBP**	**DBP**	**SBP**	**DBP**
Initial value	95.47 ± 21.40^a^	81.40 ± 19.42^A^	98.38 ± 17.53^a^	67.56 ± 21.65^A^
The first week	109.10 ± 13.31^b^	84.30 ± 16.41^A^	120.89 ± 19.65^a^	88.86 ± 17.71^A^
The second week	119.42 ± 14.67^b^	95.24 ± 15.23^A^	135.22 ± 20.34^a^	88.42 ± 17.44^A^
The third week	112.19 ± 27.34^b^	94.48 ± 28.36^B^	159.84 ± 36.75^a^	113.00 ± 29.16^A^
The fourth week	139.53 ± 27.32^b^	119.92 ± 26.08^B^	188.80 ± 30.10^a^	140.71 ± 29.01^A^
The fifth week	135.13 ± 16.86^b^	104.64 ± 21.97^B^	190.92 ± 41.31^a^	140.81 ± 34.15^A^
The sixth week	149.08 ± 16.52^b^	118.32 ± 13.34^B^	190.92 ± 41.31^a^	145.76 ± 40.61^A^

*CZ, normal group, CG, hypertensive group, DBP represents diastolic blood pressure, SBP represents systolic blood pressure. 2. Upper case letters were used to compare DBP between the CZ and CG groups, lower case letters were used to compare SBP between the CZ and CG groups, different letters indicated significant differences (P < 0.05)*.

### Diversity and composition of the microbiota

#### Microbial diversity and OTU identification from phylum to species levels

**Figure 1:**
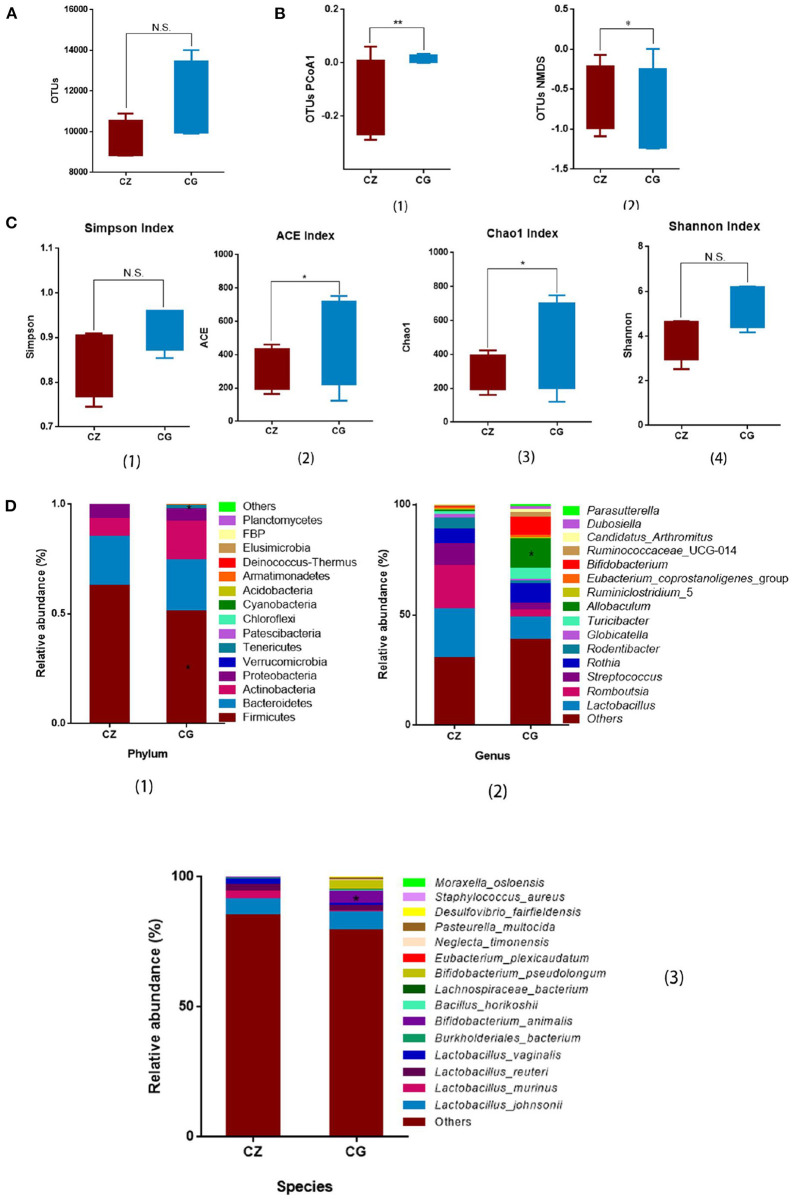
Hypertension of liver-Yang hyperactivity syndrome induced by a high salt diet alters intestinal microbiota diversity and composition. **(A)** Bar plots of observed operational taxonomic unit (OTUs). **(B)** Box plots of β - diversity index of PCoA1 and NMDS of OTUs, represented by the space distance among groups. **(C)** Bar plots of α - diversity index of OTUs (Chao1, Simpson, ACE, and Shannon). **(D)** Relative mean abundance of (1) phyla, (2) genus and (3) species in the intestinal contents, **(A–D)** *P < 0.05, **P < 0.01; N.S., not significant (*P* > 0.05). CZ, normal group, CG, hypertensive group.

The OTU number did not differ between the CZ and CG groups (Figure 1A). High salt intake significantly perturbed alpha diversity, as determined by Chao1 (*P* = 0.021) and ACE (*P* = 0.032) indices (Figure 1C 2, 3), with no significant differences in Simpson and Shannon indices between groups (Figure 1C 1, 4). Beta diversity, as evaluated by PCoA and non-metric multidimensional scaling (NMDS), also differed significantly between the CZ and CG groups (*P* = 0.009 and 0.042 for PCoA and NMDS, respectively) (Figure 1B 1, 2). Alpha diversity and beta diversity were based on OTUs.

From the phylum to species levels, bacterial communities were systematically classified and compared between the CZ and CG groups. Members of the phylum Firmicutes were the most abundant in the CZ and CG groups, accounting for 62.69 and 51.12% of OTUs, respectively, followed by members of the phyla Bacteroidetes and Actinobacteria. Firmicutes showed a significant difference in abundance between groups (Figure 1D 1, *P* > 0.05), while Bacteroidetes and Actinobacteria did not differ in abundance between the CG and CZ groups (Figure 1D 1, *P* > 0.05). The phyla Chloroflexi and Cyanobacteria were only detected in the CZ group and Armatimonadetes, Deinococcus-Thermus, FBP, and Planctomycetes were only detected in the CG group.

At the genus level, the top three genera were *Lactobacillus, Romboutsia*, and *Streptococcus;* however, there were no differences in relative abundance between groups (Figure 1D 2, *P* > 0.05). The relative abundance of *Allobaculum* in the CZ group was significantly lower than that in the CG group, and *Dubosiella* was only observed in the CG group.

At the species level, among the top 15 dominant species, the top four were all *Lactobacillus: L. johnsonii, L. vaginalis, L. murinus*, and *L. reuteri*. The frequencies of the four species did not differ significantly between groups (Figure 1D 3, *P* > 0.05). *Bifidobacterium animalis* had a significantly higher frequency in the CG group than in the CZ group. *Eubacterium plexicaudatum* (0.07% vs. 0) was unique to the CZ group, and *Pasteurella multocida* (0 vs. 0.59%) was unique to the CG group.

#### Microbial composition and key bacteria

**Figure 2:**
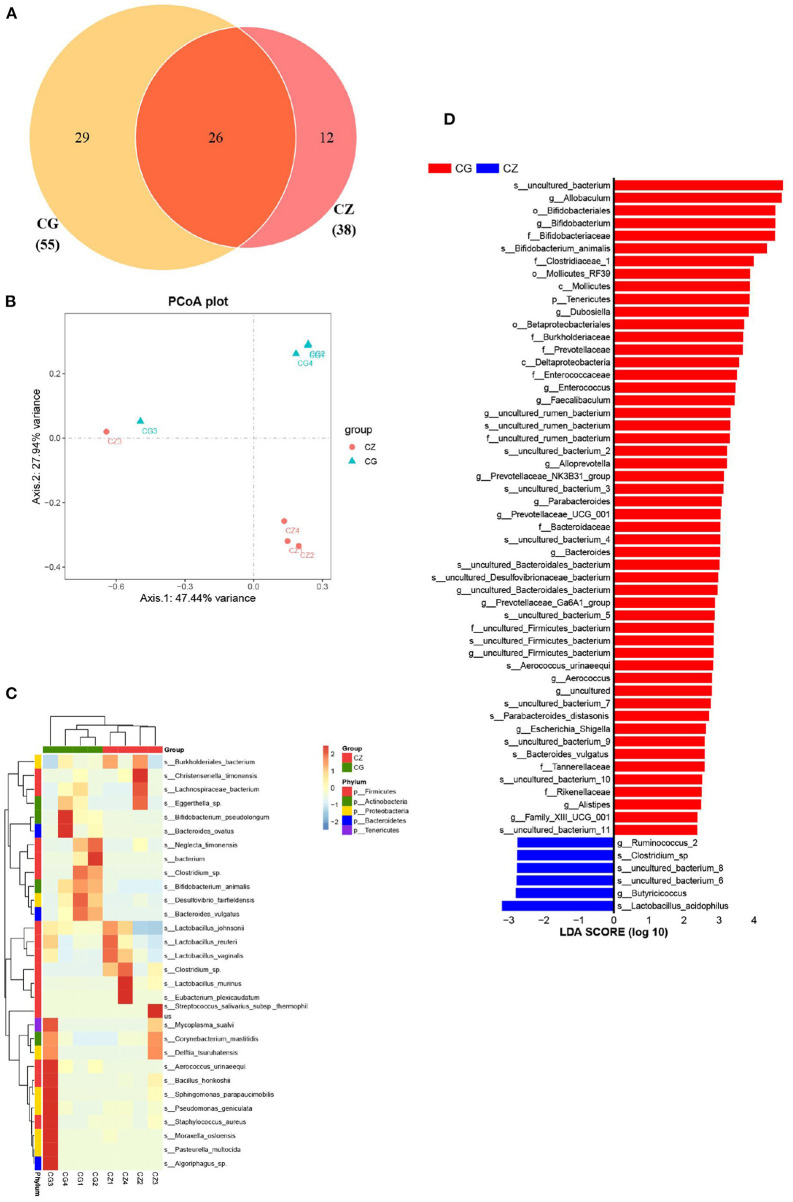
The diversity, composition and key bacteria of intestinal microbiota with hypertension of liver-Yang hyperactivity syndrome induced by a high salt diet at species level. **(A)** Venn diagram of OTU distribution at species level. **(B)** Cluser analysis of PCoA based on the OTUs of taxonomic bacterial species. **(C)** Heatmap clustering of community species abundance at species level, and the closer the color is to red, the higher the abundance. **(D)** Cladogram generated from the LEfSe analysis indicating the phylogenetic distribution from family to species of the microbiota (LDA score ≥ 2, *P* < 0.05). CZ, normal group, CG, hypertensive group.

Beta diversity and two indicators of alpha diversity, Chao1 and ACE, differed significantly between groups (Figures 1B,C). Our study of the gut bacterial species involved in hypertension induced by a high salt diet was largely based on taxon abundance, rather than OTUs. Based on analyses of relative abundance from the phylum to species level, the composition of the gut microbiota differed between groups (Figure 1D 1–3). A Venn diagram of the species level results revealed 55 and 38 OTUs in the CZ and CG groups, with 26 shared OTUs (Figure 2A). Additionally, based on the OTUs of taxonomic bacterial species, 75.38% of the total variance was explained by principal components 1 and 2 in the clustering analysis and the PCoA diagram showed obvious separation between groups (Figure 2B).

A heatmap of community species abundance at the species level revealed that the top 30 species in the CZ and CG groups were dominated by Firmicutes, followed by Proteobacteria (Figure 2C). In the LEfSe analysis, the two groups had a significant structural difference and 58 signature bacterial taxa differentiated the groups (LDA Score > 2) (Figure 2D). Excluding the unculturable taxa, we identified 8 species, 14 genera, and 8 families with differences in abundance. The LEfSe analysis showed that *Lactobacillus acidophilus, Clostridium* sp.*, Parabacteroides distasonis, Bifidobacterium animalis, Parabacteroides johnsonii, Aerococcus urinaeequi*, and *Bacteroides vulgatus* were identified as key discriminant taxa. The four species *Parabacteroides johnsonii, Parabacteroides distasonis, Bacteroides vulgatus*, and *Aerococcus urinaeequi* were found to be only present in the CG group. *Lactobacillus acidophilus* and *Clostridium* sp. were found to be only present in the CZ group. The species identified in this analysis might be indicators of hypertension induced by high salt intake.

### Intestinal metabolite analysis and key differential metabolites

**Figure 3:**
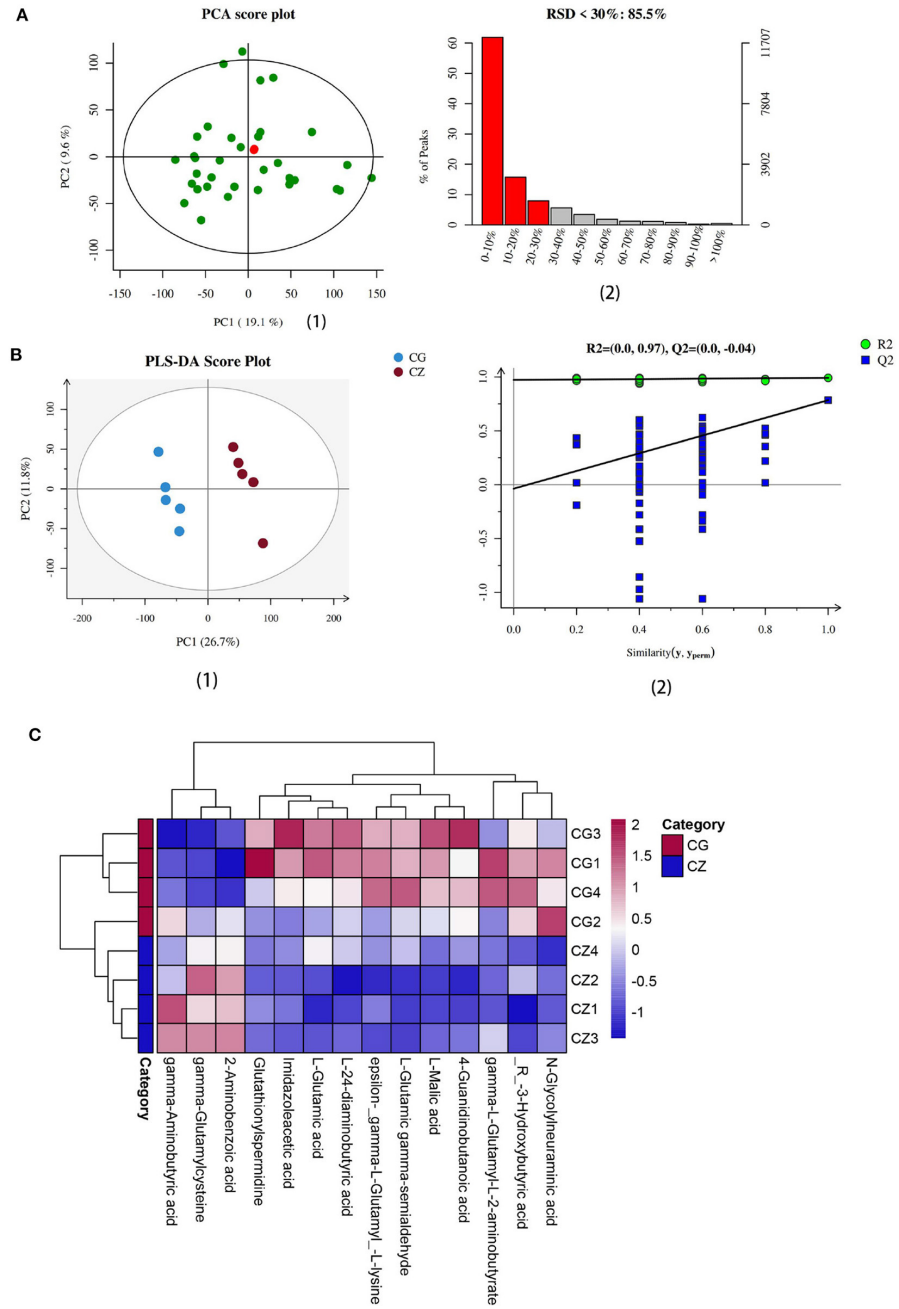
Determination and heat map of intestinal metabolites we focus in hypertension of liver-Yang hyperactivity syndrome induced by a high salt diet. **(A)** Quality assurance results diagramof positive ions. (1) The middle green dots are samples (the figure is measured together with multiple groups of data, two of which are used in this paper), the red dots are QC samples. (2) The left ordinate is the proportion of RSD <30%, the right ordinate is the specific value, and the abscissa is the range of RSD value. **(B)** (1) PLS-DA score plot of positive ions, (2) Permutation testing of positive ions**. (C)** The heat map of cluster analysis of differential metabolites. CZ, normal group, CG, hypertensive group.

**Figure 4:**
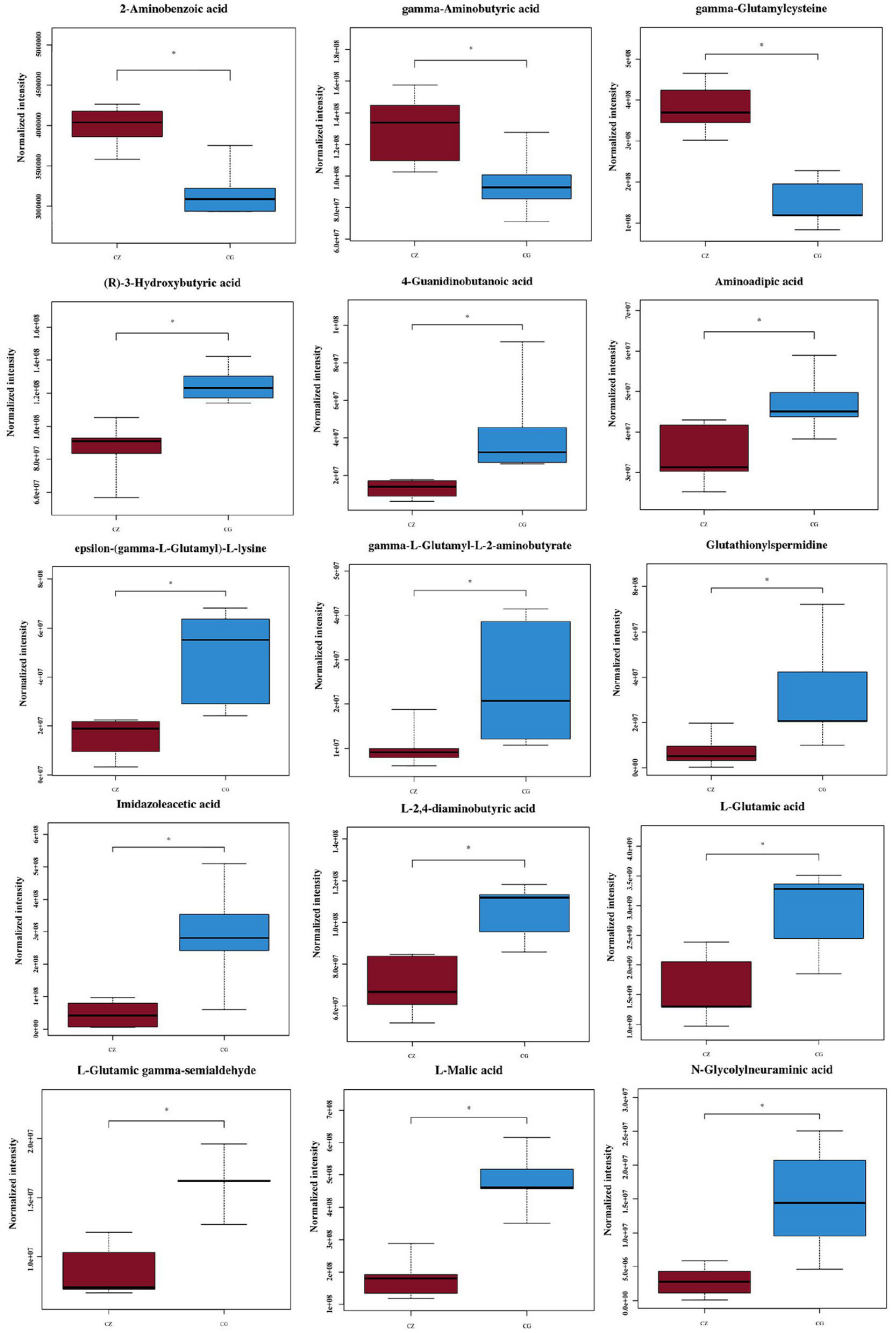
Fifteen significantly different metabolites we focus of hypertension of liver-Yang hyperactivity syndrome induced by a high salt diet. CZ, normal group, CG, hypertensive group. *P < 0.05.

Intestinal metabolites vary with the gut microbiota. We evaluated intestinal metabolites in the CZ and CG groups. The total ion current (TIC) chromatograms of typical LC/MS for non-targeted metabolomics in positive and negative modes revealed an obvious difference between the two groups. As shown in **Figure 4A**, the ratio of characteristic peaks with RSD <30% was 85.5% and *R*^2^ was 0.595, indicating that the detection data were reliable. PLS-DA showed that the two groups could be separated and showed a significant difference in metabolite profiles (Figures 3A,B 1, 2).

We used VIP ≥ 1, *P* ≤ 0.05, and one-way ANOVA *P* ≤ 0.05 to screen metabolites (26, 27). We found that the differential metabolites were primarily short chain fatty acids (mainly butyric acid), amino acids (mainly glutamic acid), and their derivatives (Figure 4). Among 15 metabolites shown in Figure 4, levels of 3 metabolites in the CZ group were significantly higher than those in the CG group (*P* < 0.05); in contrast, levels of 12 metabolites were higher in the CG group than in the CZ group (*P* < 0.05). A heat map tree of the clustering analysis (Figure 3C) were constructed to visualize the 15 differential metabolites. The contents of gamma-aminobutyric acid and 2-aminobenzoic acid were significantly lower in the CG group and the contents of glutamic acid and its derivatives (e.g., glutathionylspermidine, L-glutamic acid, L-glutamic gamma-semialdehyde, epsilon-(gamma-L-Glutamyl)-L-lysine and gamma-L-glutamyl-L-2-aminobutyrate) were significantly higher in the CG group than in the CZ group.

### Differential metabolite pathways

**Figure 5:**
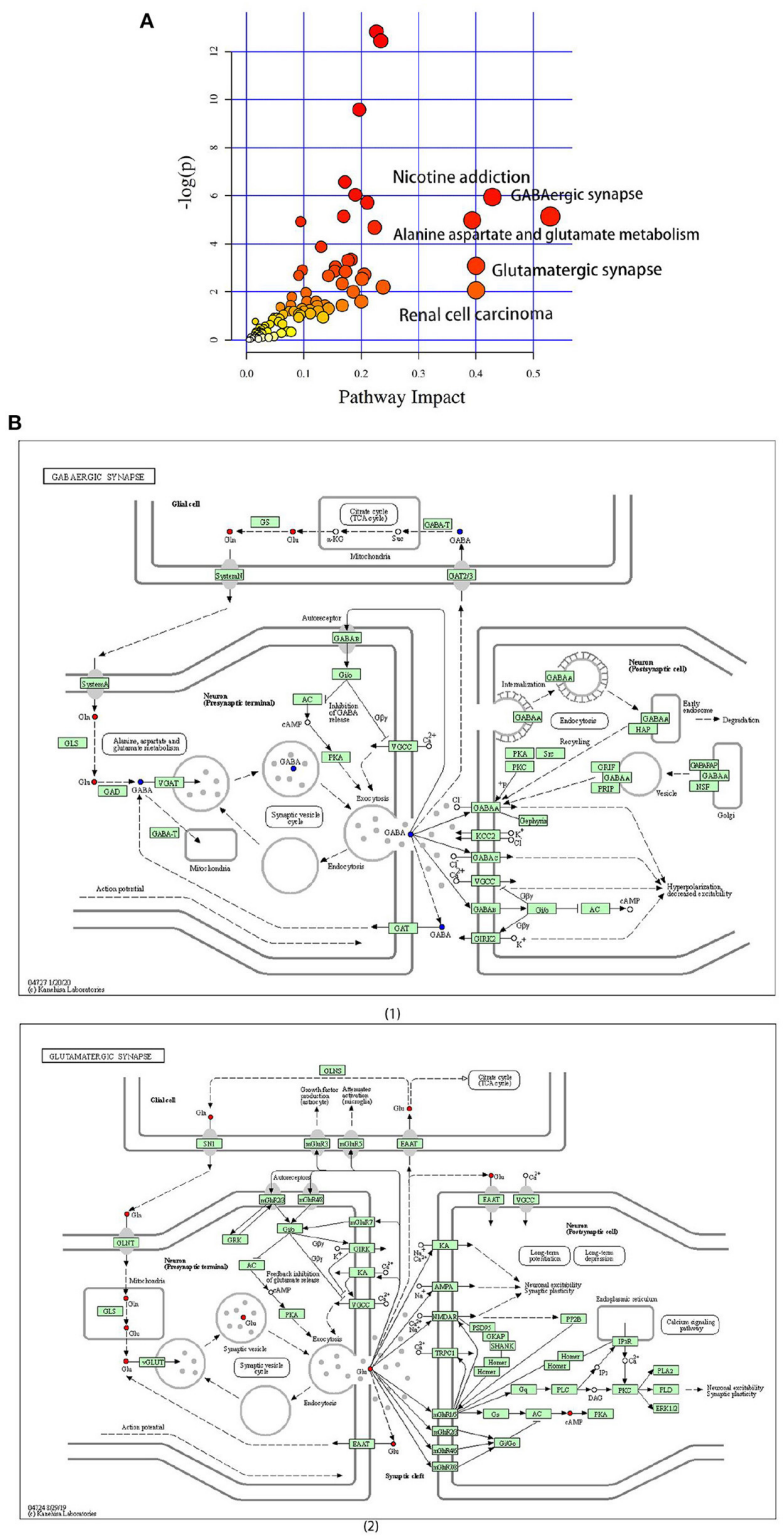
The significantly different metabolites enriched KEGG pathways analysis. **(A)** Pathways of differential metabolites, the size and color are based on the *p*-value and impact value, small *p*-value and big pathway impact value indicate that the pathway is greatly influenced. **(B)** (1) Overall perspective of GABAergic synapse pathway metabolism map; (2) Overall perspective of Glutamatergic synapse pathway metabolism map. In the overall perspective of pathway map, red represents increased and blue represents decreased.

A pathway enrichment analysis of significantly dysregulated metabolites was performed. On the basis of the impact value and *P*-value, we identified the five most influential metabolic pathways (Table 2 and Figure 5A). The GABAergic synapse pathway (impact value = 0.529) was the most relevant pathway, followed by the nicotine addiction pathway (impact value = 0.429), glutamatergic synapse pathway (impact value = 0.4), renal cell carcinoma (impact value = 0.400), and alanine, aspartate, and glutamate metabolism (impact value = 0.393). These five pathways were consistently related to GABA and the glutamate/glutamine metabolism. In particular, the GABA content was lower and glutamic acid and its derivatives content was higher in the CG group than in the CZ group (Figure 5B 1, 2).

**Table 2:** The top five differential metabolite pathways.

**Pathway name**	**Total**	**Hits**	**Raw p**	**-log(p)**	**Holm adjust**	**FDR**	**Impact**
GABAergic synapse	9	3	0.0058928	5.134	1	0.17875	0.529
Nicotine addiction	7	3	0.002622	5.9438	0.7027	0.1193	0.429
Glutamatergic synapse	8	2	0.04587	3.0819	1	0.76927	0.400
Renal cell carcinoma	3	1	0.12726	2.0616	1	1	0.400
Alanine, aspartate and glutamate metabolism	8	1	0.30459	1.1888	1	1	0.393

*Total: the total number of compounds in the pathway. Hits: the number of differential metabolites in target metabolic pathways. Raw p: p value of hypergeometric distribution test. The original p value calculated from the enrichment analysis. -log(p): take the negative value of the natural log of the p value; Holm adjust p: the p value adjusted by Holm–Bonferroni method. FDR p: p value adjusted using False Discovery Rate. Impact: the pathway impact value calculated from pathway topology analysis*.

### Correlations between the gut microbiota and metabolite profile

The composition of the gut microbiota is in a dynamic state and is affected by diet, and intestinal metabolites vary with the gut microbiota (28, 29). For the CZ and CG groups, as we described earlier, we identified 8 species, 14 genera, and 8 families as key discriminants. To explore the associations between the relative abundances of these key discriminant taxa and concentrations of 15 differential metabolites we focus, we performed correlation analyses based on Spearman correlation coefficients.

**Figure 6:**
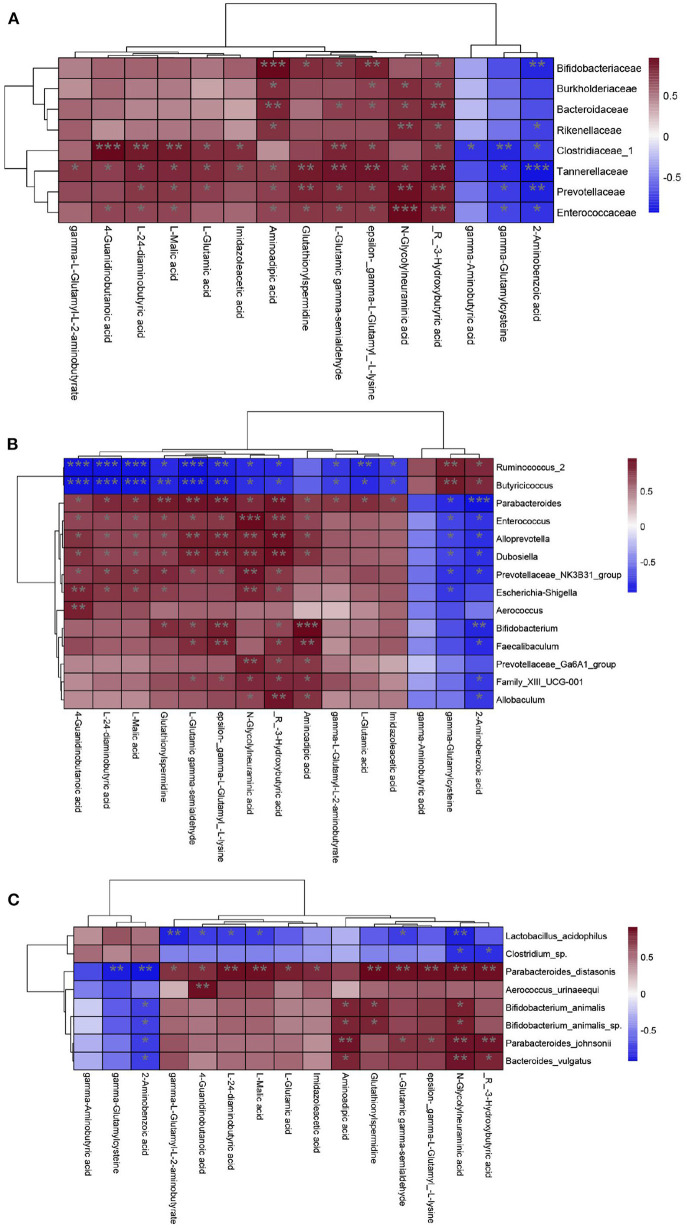
Correlation analysis of gut microbiota and metabolites from family to species level. **(A)** Family level. **(B)** Genus level. **(C)** Species level. Heat maps indicated positive (red) and negative (blue) correlations between the dominant species and 15 differential metabolites we focus. The legend shows correlation values from −1 to 1 and assigns the appropriate color to them (Red for positive correlations and blue for negative correlations). **(A–C)** *P < 0.05, **P < 0.01.

The KEGG analysis revealed that differential metabolites between the CZ and CG groups were enriched for pathways related to GABA and glutamate/glutamine metabolism; accordingly, these were the focus of subsequent analyses. GABA was decreased and Glu was increased in the CG group compared with the CZ group. At the species level, *Parabacteroides distasonis* was significant positively correlated with glutamic acid and its derivatives metabolism (*P* < 0.05) (Figure 6C). At the genus level, *Parabacteroides* was significant positively correlated with glutamic acid and its derivatives metabolism (*P* < 0.05) (Figure 6B), while significant negative correlations were observed for *Ruminococcus_2* and *Butyricicoccus*. At the family level, Clostridiaceae_1 was significantly negatively correlated with GABA metabolism and positively correlated with glutamic acid and its derivatives metabolism (*P* < 0.05) (Figure 6A).

## Discussion

The occurrence of hypertension is influenced by many factors, including environmental and dietary factors. There is substantial evidence that long-term high salt intake could raise blood pressure; however, the mechanism underlying this effect is unclear. When food enters the digestive tract orally, it inevitably interacts with the intestinal microbiota. The digestion and absorption of food depends on decomposition and metabolism by the gut microbiota (30–32). In addition, changes in intestinal microecology in response to ingested substances determine nutrient absorption to a certain extent (33). Diet changed the gut microbiota composition, structure, and metabolites, some of which can be absorbed into the circulation and affect host health, with the potential to result in hypertension or heart failure (34–36). Probiotic effector molecules were particularly important for the rugulation of gut microbiota (37). The roles of interactions between the host, gut microbiota, and metabolites in the pathogenesis of hypertension have become a focus of research (38, 39). These interactive effects prompted questions regarding which components of the gut microbiota and metabolites and which metabolic pathways are altered by a high salt diet to elevate blood pressure. Our results address this question. We diagnosed the TCM syndrome of hypertension induced by a high salt diet and determined that the hypertension induced by a high salt diet belonged to the liver-Yang hyperactivity syndrome. Based on 16S rRNA SMRT gene full-length sequencing and UPLC-MS, we analyzed the bacterial species composition, biodiversity, and differential metabolites between the CG and CZ groups. We provide the first evidence that a high salt diet inhibits the growth of Clostridiaceae_1 and affects the glutamate/GABA-glutamine metabolic cycle, thereby promoting the increase in blood pressure.

According to the expert consensus on diagnosis and treatment of hypertension with TCM, the clinical symptoms of hypertension of liver-Yang hyperactivity syndrome were dizziness, tinnitus, headache, irritability, etc. These kinds of clinical symptom transformed to the rat were the degree of resistance to capture increased, and the spirit is excited and so on (4). Hypertensive rats induced by a high salt diet showed the typical characteristics of liver-Yang hyperactivity syndrome, such as impatience and irritability. The reason is that hypertension is greatly affected by environmental and dietary factors. The mice in CG group lived in the same environment and have the same dietary structure. High salt diet caused increased drinking water and urine output, so as to caused kidney burden. We could be sure that hypertension induced by a high salt diet is the syndrome of liver-Yang hyperactivity.

We found that the Chao1 and ACE indexes of alpha diversity were significantly increased in hypertensive rats, with no obvious differences in beta diversity between CZ and CG groups. Combined with our PCoA and NMDS results, a high salt diet clearly changed the structure of the gut microbiota, and intestinal bacteria may show increased growth in hypertensive animals. In fact, many recent studies have reported that increased growth of gut bacteria is common in patients with a variety of diseases (40, 41).

At the phylum level, the relative abundance of Firmicutes was higher in the CZ group than in the CG group, while the opposite pattern was observed for Tenericutes. The abundance of Bacteroidetes in the CG group was higher than that in the CZ group; however, the difference was not significant. The observed differences at the phylum level were consistent with previous results indicating that a decrease in Firmicutes and increase in Bacteroidetes are associated with hypertension (42, 43). At the species level, the *P. distasonis* was only present in the CG group and was significant positively correlated with glutamate/glutamine metabolism. *P. distasonis* had been proved to be closely related to modulate host metabolism, such as produced secondary bile acids and succinate to alleviate obesity and metabolic dysfunctions (44). We did not detect associations between bacterial species and GABA metabolism, which may be explained by the unculturable species identified in the analysis (Figure 2). At the family level, the abundance of Clostridiaceae_1 was significantly negatively correlated with GABA metabolism and positively correlated with glutamate/glutamine metabolism (*P* < 0.05). These results suggested that high salt intake inhibits the growth of Clostridiaceae_1, which might be contribute to hypertension.

GABA and Glu(or derivatives) were the key differential metabolites identified in this study. The GABA content was significantly decreased and Glu(or derivatives) content increased in CG group compared with the CZ group. These changes can induce hypertension. Glutamate/GABA-glutamine cycle is associated with movement of ammonia nitrogen between the two cell types and pyruvate carboxylase, enzymes glutamine synthetase and phosphate-activated glutaminase were the key enzyme required for this cycle (45). GABA is a non-proteinogenic amino acid found in plants, vertebrates, and microorganisms. GABA is involved in the synaptic transmission modulation and neuronal development, among other physiological processes (46). GABA is widely distributed in different regions of the brain and is a main inhibitory neurotransmitter in the central nervous system (47, 48). Glutamate and glutamine are precursors of GABA production. Bacteroides plays a very important role in the regulation of the GABAergic system in the human gut, and the gut microbiota could produce GABA, thereby modulating the gut–brain axis (49, 50). GABA-salt could reduce hypertension by reducing endothelial cell dysfunction and M1 polarization (51). On non-neuronal peripheral tissues and organs, GABA has intestinal protective, anti-microbial, and anti-hypertensive properties (52, 53). Glutamatergic and GABAergic signals are related to neurotransmitter vesicle loading, signal inactivation, and neurotransmitter supplementation in the central nervous system (54, 55). In the brain, Glu and GABA are the main excitatory and inhibitory amino acids, and they can modulate the neuronal excitation of the rostral ventrolateral medulla and the firing activity of blood pressure-related neurons (56). GABA can drive sympathetic tone to inhibit the activity of the glutamatergic neurons directly (57). It is released from local GABAergic interneurons to mediate the hypertensive response, and the GABAergic system is related to Na(+)-dependent hypertension (58, 59). Our results suggested that high salt induces hypertension *via* pathways associated with GABA and glutamate/glutamine metabolism.

## Conclusion

Our results provide novel insight into the microbial mechanisms underlying hypertension induced by a high salt diet and provide a basis for the TCM diagnosis and treatment of hypertension. Hypertension induced by a high salt diet belonged to the liver-Yang hyperactivity syndrome and high salt intake altered the gut microbiota, inhibited the growth of Clostridiaceae_1, and affected GABA metabolism-related pathways, resulting in GABA and glutamate/glutamine metabolic disorders and thereby increasing blood pressure. GABA is distributed in a wide range of brain regions. Accordingly, it is a key neurotransmitter by which the bacterial community contributes to the brain–gut interaction. Our results suggested that a high salt diet induced hypertension of liver-Yang hyperactivity syndrome by mediating the microbiota associated with the glutamate/GABA-glutamine metabolic cycle *via* the gut–brain axis. Next step, we can focus on the relationship between TCM treatment of hypertension induced by high-salt diet and gut microbiota and metabolites, which can provide a basis for TCM treatment of hypertension.

## Data availability statement

The data presented in the study are deposited in the (NCBI SRA) repository, accession number (PRJNA822435).

## Ethics statement

The animal study was reviewed and approved by Animal Ethics and Welfare Committee of Hunan University of Chinese Medicine.

## Author contributions

TZ, Z-jT, and TY conception and design of research. TZ, YW, and M-jP performed experiments, data analysis, and data interpretation. N-qX drafted the manuscript. All authors contributed to the article and approved the submitted version.

## Funding

Financial support was received from the following project: Innovation and entrepreneurship training program for college students of Hunan Province, China (S202010541049), Open fund of Key Laboratory of Agro-Products Processing, National Risk Assessment Laboratory of Agro-products Processing Quality and Safety, Ministry of Agriculture and Rural Affairs, P. R. China (S2020KFKT-18), and Hunan Provincial Science and Technology Department, China (2020SK2140).

## Conflict of interest

The authors declare that the research was conducted in the absence of any commercial or financial relationships that could be construed as a potential conflict of interest.

## Publisher's note

All claims expressed in this article are solely those of the authors and do not necessarily represent those of their affiliated organizations, or those of the publisher, the editors and the reviewers. Any product that may be evaluated in this article, or claim that may be made by its manufacturer, is not guaranteed or endorsed by the publisher.
